# Essential Mycoplasma Glycolipid Synthase Adheres to the Cell Membrane by Means of an Amphipathic Helix

**DOI:** 10.1038/s41598-019-42970-9

**Published:** 2019-05-08

**Authors:** Javier Romero-García, Xevi Biarnés, Antoni Planas

**Affiliations:** Laboratory of Biochemistry, Institut Químic de Sarrià, Universitat Ramon Llull, Barcelona, Spain

**Keywords:** Glycobiology, Protein structure predictions

## Abstract

Because of the lack of cell wall, *Micoplasma* species require a fine control of membrane fluidity and integrity. *mg517* is an essential gene of *Mycoplasma genitalium* responsible for the biosynthesis of membrane glycoglycerolipids. It encodes for a unique glycosyltransferase (MG517) with processive activity, transferring activated glycosyl donors to either nude diacylglycerol or already glycosylated diacylglycerol. This dual activity, asserted to different enzymes in other species, is sensitive to and regulated by the presence of anionic lipid vesicles *in vitro*. We present here a computational model of the C-terminus domain of MG517 that complements a previous structural model of the N-terminus domain. By means of sequence analysis, molecular dynamics and metadynamics simulations, we have identified a short α-helix at the apical C-terminus of MG517 with clear amphipathic character. Binding to a membrane model is thermodynamically favored which suggests that this structural element guides the adhesion of MG517 to the cell membrane. We have experimentally verified that truncation of part of this helix causes a substantial reduction of glycoglycerolipids synthesis. The model proposes that MG517 recognizes and binds the diacylglycerol substrate embedded in the membrane by means of this α-helix at the C-terminus together with a previously identified binding pocket at the N-terminus.

## Introduction

Membrane proteins can either be “peripheral” or “integral” depending on how they associate to the lipid bilayer^1^. From one hand, peripheral membrane proteins bind only temporarily at one side of the membrane (monotopic interaction) or are bound to other proteins by weak noncovalent interactions at the membrane interface. The reversible attachment of peripheral membrane proteins has shown to regulate cell signaling and many other important cellular events^2^. Such a protein-membrane association can be easily disrupted with the use of alkaline and high ionic strength buffers that leave the lipid bilayer intact^1^. On the other hand, the binding to the membrane of integral membrane proteins is strong and permanent, usually adopting structural functions such as transporters, linkers, channels and proteins responsible for cell adhesion^3^. Integral membrane proteins can only be extracted from the membrane by disrupting the lipidic bilayer, using either detergents or organic solvents^4,5^. Depending on the way integral membrane proteins are embedded in the membrane, these are termed monotopic, bitopic or polytopic proteins^6^. Monotopic integral membrane proteins associate permanently to only one face of membrane. Bitopic and polytopic integral membrane proteins exhibit one or more transmembrane segments that cross the membrane at different levels^7,8^.

The 1,2-diacylglycerol 3-glucosyltransferase from *Acholeplasma laidlawii* (*Al*MGS), a cell wall-less prokaryote, is a good representative of monotopic enzymes, from which the molecular details of peripheral protein-membrane binding can be drawn^8^. The charge density and curvature properties of the *A. laidlawii* membrane is controlled by this monoglucosyldiacylglycerol synthase (*Al*MGS) and the diglucosyldiacylglycerol synthase (*Al*DGS), which are associated to the cyosolic side of the membrane. These two glycolipid synthases are glycosyltransferases (GT) that catalyze the transfer of glucosyl residues from uridine diphosphoglucose (UDPGlc) to a diacylglycerol (DAG) acceptor, sequentially synthesizing monoglucosyl diacylglycerol (GlcDAG) and diglucosyl diacylglycerol (GlcGlcDAG), respectively^9^. The molar ratio between these two glucolipids at the membrane affects its curvature and is adjusted by the activity of these two enzymes, which is altered by different stimuli, including ionic strength and aliphatic chains composition. The use of detergents are required to extract *Al*MGS from the membrane, albeit no clear hydrophobic segments that span the membrane are identified^10^. The C-terminus domain of *Al*MGS is predominantly acidic, whereas the N-terminus domain is basic^11^. An amphipathic α-helix at the N-terminus, mainly composed of a combination of hydrophobic residues and arginine and lysine amino acids, has been identified by different biophysical techniques^12^. Based on these observations, a model of protein-membrane interaction was proposed in which the protein is irreversibly attached by means of an amphipathic helix^10^, as seen in many other proteins^13,14^. This type of monotopic association to the membrane is also reported for the recently crystallized galactosyl diacylglycerol synthase, the MGD1 from *Arabidopsis thaliana* (PDB codes 4WYI and 4 × 1T). This protein does not bind to the membrane by means of an amphipathic helix, but with a long disordered region which contains alternated hydrophobic and hydrophilic regions^15^.

The processive diacylglycerol β-glycosyltransferase from *Mycoplasma genitalium* (MG517)^16–18^ shares many of the characteristics described for *Al*MGS. Both belong to organisms classified in the Mollicutes class^19^, where the lack of cell wall requires a fine regulation of the membrane fluidity and integrity. As opposed to *A. laidlawii* that has two different glycosyltransferases (GT) for mono- and di-synthase activities, *M. genitalium* GT MG517 is a processive (or sequentially acting) GT able to transfer the sugar to either a diacylglycerol or a monoglycosyldiacylglycerol acceptor^18^, thus requiring other mechanisms to regulate the balance between non-bilayer and bilayer-prone glycolipids. It is activated by anionic phospholipids and requires anionic lipid vesicles for *in vitro* activity experiments, suggesting that protein-membrane interactions not only are required for protein activation but also to regulate its processive behavior^16^. GT MG517 belongs to family 2 of glycosyltransferases (GT2) in the CAZY database (carbohydrate active enzymes, www.cazy.org ^20^, a family of proteins that shares a GTA fold, in contrast with *Al*MGS and *Al*DGS that show a GTB fold and belong to family GT4. Structural information of GT MG517 is scarce due to failed attempts of crystallization. We reported a partial computational model of the structure which comprises the N-terminus region of the protein (residues 1 to 220)^17^. That computational model confirmed the GTA fold and allowed the identification of key residues for catalysis and a flexible region involved in acceptor binding, which enabled the first steps towards rational drug design, since *M. genitalium* is a well-known human pathogen^19^. However, the structure of the C-terminus region of MG517 still remains unknown (residues 221 to 341). MG517 can be purified without the use of detergents, but the purified enzyme retains membrane lipids that can only be released when the enzyme is extracted with detergents^18^. This suggests a soft binding of the enzyme with the *M. genitalium* membrane that could be monotopic due the lack of hydrophobic trans-membrane segments. Expression of the N-terminus fragment of GT MG517 (residues 1 to 220) did not require the use of detergent to release membrane lipids^18^, suggesting that membrane binding may occur in some part of the C-terminus region.

We here propose a membrane binding strategy for GT MG517, which involves an apical amphipathic helix located at the C-terminus. The computational strategy here reported may be of general application to identify, characterize and quantify the interaction of amphipathic helices with membranes in other proteins.

## Results and Discussion

### The C-terminus region of GT MG517 has no similarity with other GTs but contains a key amphipathic helix

**Figure 1:**
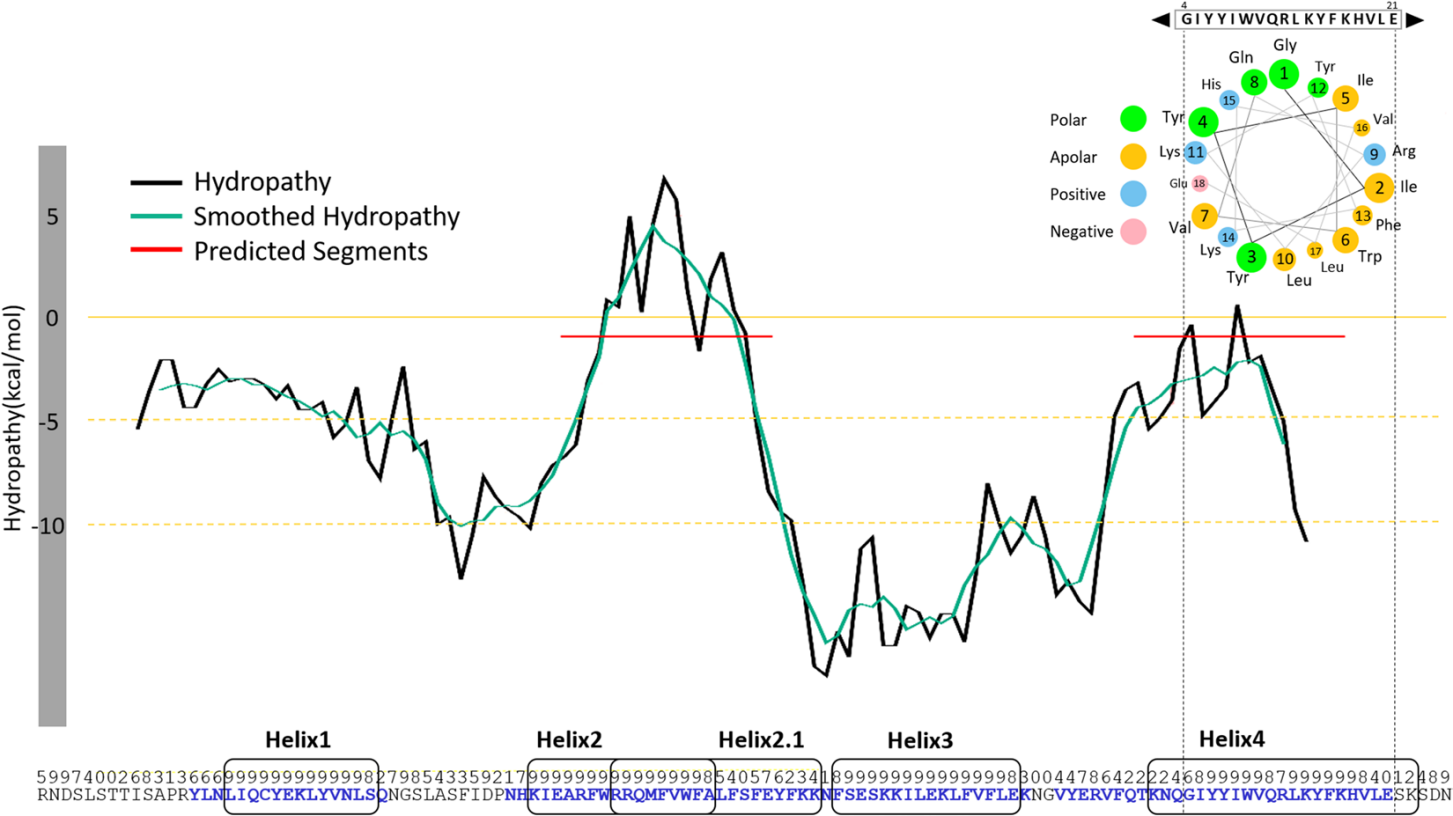
Sequence analysis of the C-terminus segment of MG517 (R221-N341). Hydropathy profile predicted with MPEx. Sequence segments with smoothed predicted hydropathy values higher than -1 kcal/mol are considered as putative membrane interaction structures (red line). Below, sequence and secondary structure prediction scores with PSI-Pred. Predicted α-helices or coiled structures are shown in blue or black letters respectively. Sequence segments selected for further structural modelling as putative membrane interaction α-helices are highlighted in round rectangles. Top-right, helical wheel representation of α-helix 4 for a window of 17 residues.

Initial attempts in the search of a proper structural template to model the MG517 C-terminus sequence (residues 221–341) were done with PSI-BLAST^21^ and HHPred^22^, with unsuccessful results. Structure predictions with ROBETTA^23^ rendered different models with no clear preference due to the lack of a structural reference (Supplemental Information, Fig. S1). We then used the PsiPred server to obtain a secondary structure prediction of the C-terminus region. The model predicted five α-helices (Figs 1 and S2), none of them corresponding to be a transmembrane helix. The hydropathy profile of the C-terminus sequence was also analyzed with the MPEx software^24^ and two sequence segments were identified as probable for membrane binding due to their hydropathy and hydrophobic properties (Fig. 1). The first region (A266-F285) has similarities to a translocon signal^25^, whereas the second region (K316-E336) has amphipathic character. The C-terminus sequence was also analyzed using an on-line helical wheel Java applet^26^. Only one helix in the apical end was found to fulfil amphipathic characteristics (Fig. 1). This comprises a 23-residue segment (K316-K338) that includes the most apical helix predicted by PsiPred and the second element predicted by MPEx as a possible membrane interaction site. The results of these predictions were combined to generate the structure, by *de novo* modelling (see methods), for five independent helices that span the whole C-terminus sequence of MG517 (Fig. 1).

**Figure 2:**
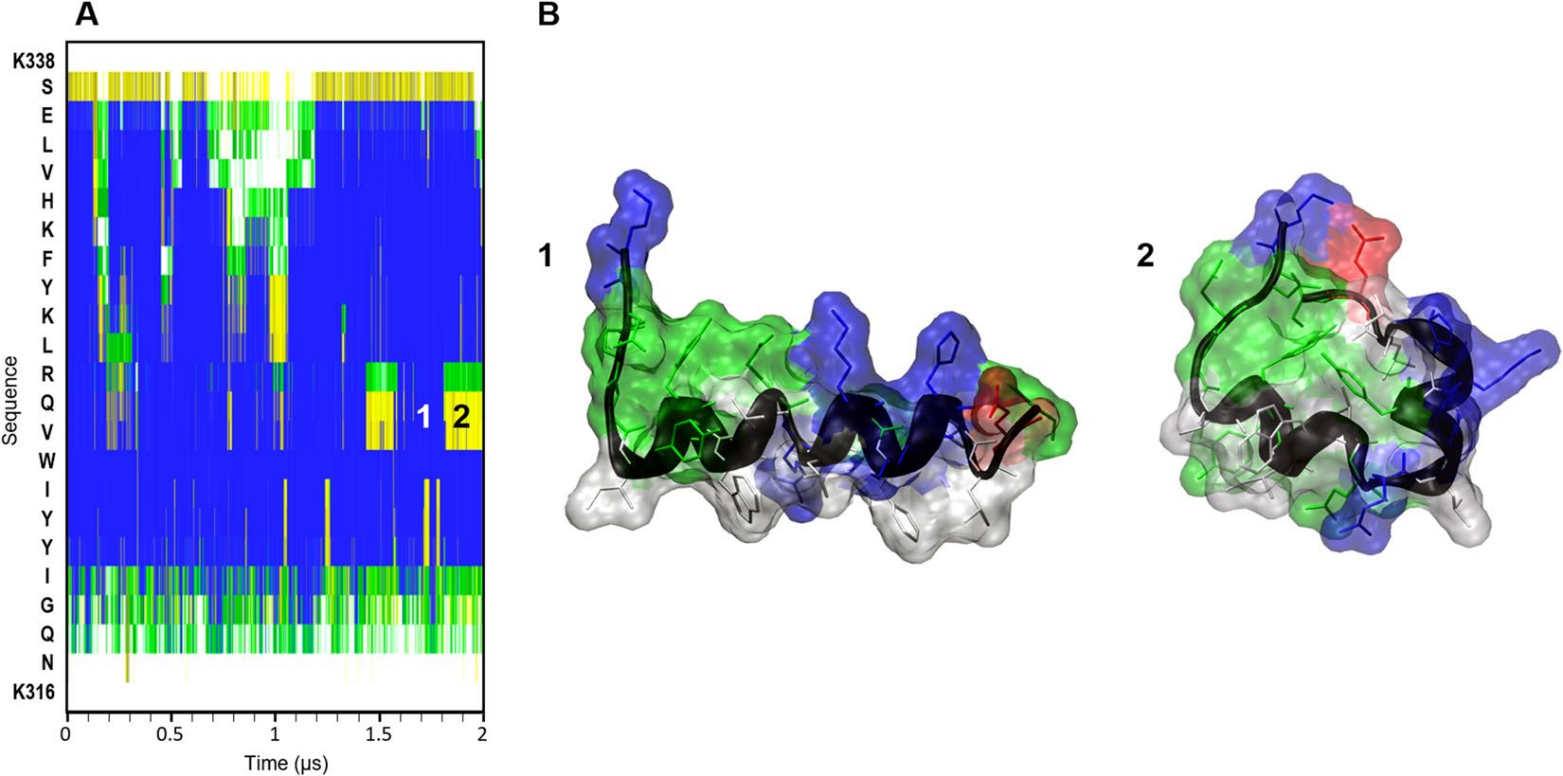
Molecular dynamics simulation of α-helix 4 of MG517 (K316-K338) in explicit solvent. (**A**) Evolution of secondary structure along the sequence (y-axis) during the simulation, computed with DSSP^37^. Blue: α-helix, yellow: turn, green: bend. (**B**) Snapshots of the structure of α-helix 4 at different simulation times: 1.7 μs (1) and 1.8 μs (2). Solvent molecules are not shown for clarity.

Molecular Dynamics (MD) simulations in aqueous solvent were performed for each of these five helices separately. Only α-helix 4 kept the fold along the whole MD (Fig. 2). The rest of the helices were unfolded with different unfolding rates (Fig. S3). Helixes 1, 2, 2.1, and 3 lost their initial α-helical conformation, and only small segments maintained the initial conformation (less than 20% of the structure was conserved at best). On the other hand, α-helix 4 kept the α-helical conformation in 75% of its sequence, which corresponds to the central core. Furthermore, α-helix 4 showed two main folded states during the last microsecond of the MD simulation (Fig. 2), one with an α-helix-like extended fold which was the major conformation, and another with a bended fold that partially covers the hydrophobic residues from the solvent, using the central residues V325, Q326 and R327 as a hinge. Since α-helix 4 is the only one with an amphipathic disposition of the amino acid residues and showed the behavior we expected for this type of element in aqueous solution, we focused on this helix to validate the amphipathic behavior in an explicit membrane environment and characterize and quantify the interaction with the membrane.

### Interaction of the amphipathic C-terminus helix with membrane models

**Figure 3:**
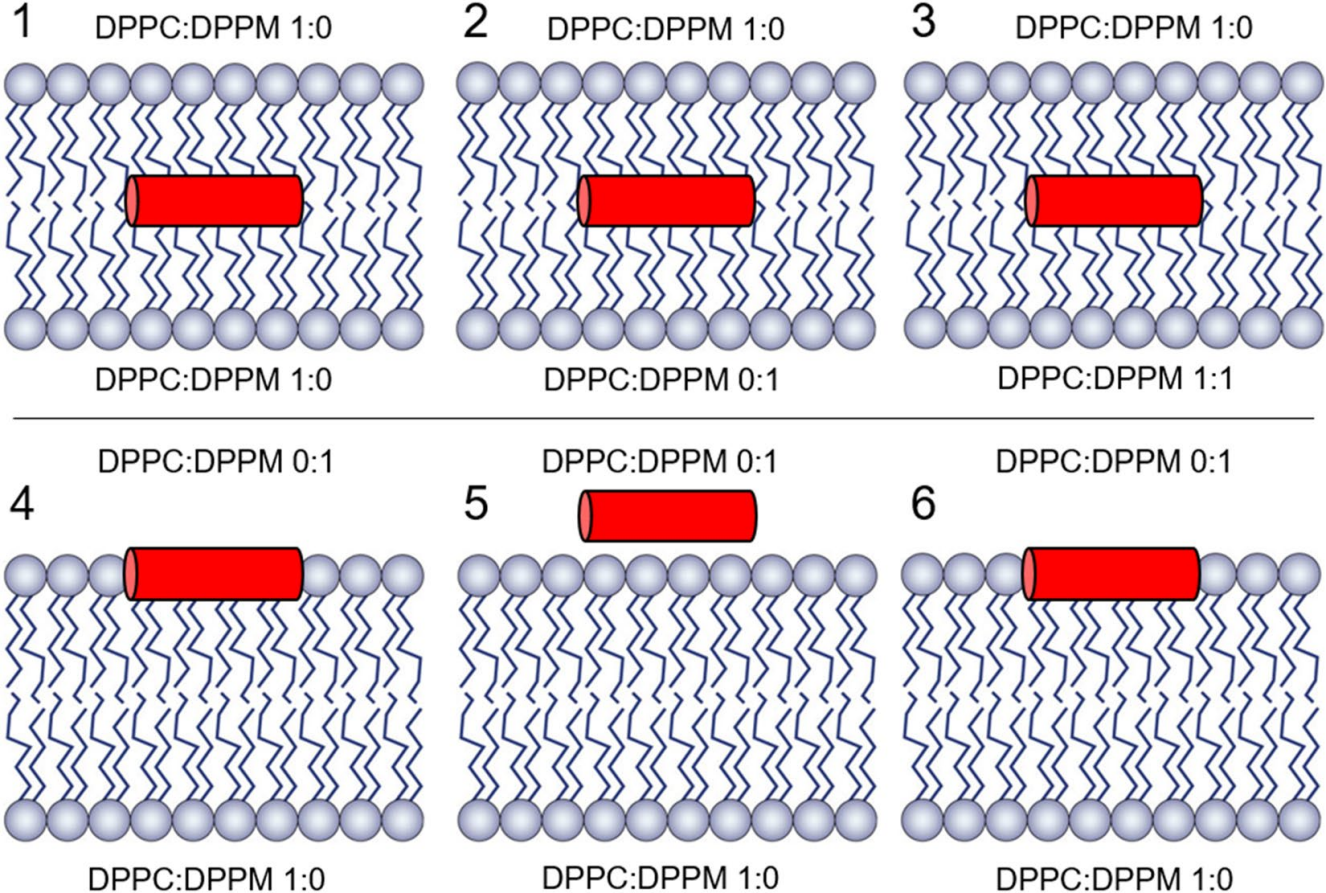
Helix 4-membrane systems. Different allocation of α-helix 4 with respect to the membrane with different charges for the lower hemimembrane (systems 1, 2, 3) and upper hemimembrane (systems 4, 5, 6) according to the DPPC:DPPM ratio as indicated: Systems 1–3, the α-helix 4 is placed in the bilayer mass center. System 4, the upper hemimembrane is forced to cover an initially static α-helix 4. System 5, the helix is placed in the solvent, at electrostatic interaction distance to the polar lipids moieties. System 6, the α-helix 4 is surrounded by the lipids as in system 4, but now with a softer packaging methodology (the same as in system 1).

To evaluate the stability of α-helix 4 in different membrane environments, six new MD simulations were performed in which a representative structure of α-helix 4, extracted from previous MD in solution, was embedded in different membrane models of varying composition. All membrane models were based on a lipid bi-layer composed of glycerophospholipids, an abundant family of lipids naturally present in the membranes of mycoplasma species^27^. Two types of lipids with different charge were considered: dipalmitoyl phosphatidylcholine (DPPC, neutral zwitterionic) and dipalmitoyl phosphatidylmethane (DPPM, anionic). Bi-layered membranes were built with different DPPC:DPPM ratios on each hemi-membrane side, generating both anionic and zwiterionic layers with varying charge densities (see Fig. 3). α-Helix 4 was placed in different positions and orientations with respect to the membrane (models 1 to 6) and different lipid packaging procedures were used before starting the simulations (see methods).

**Figure 4:**
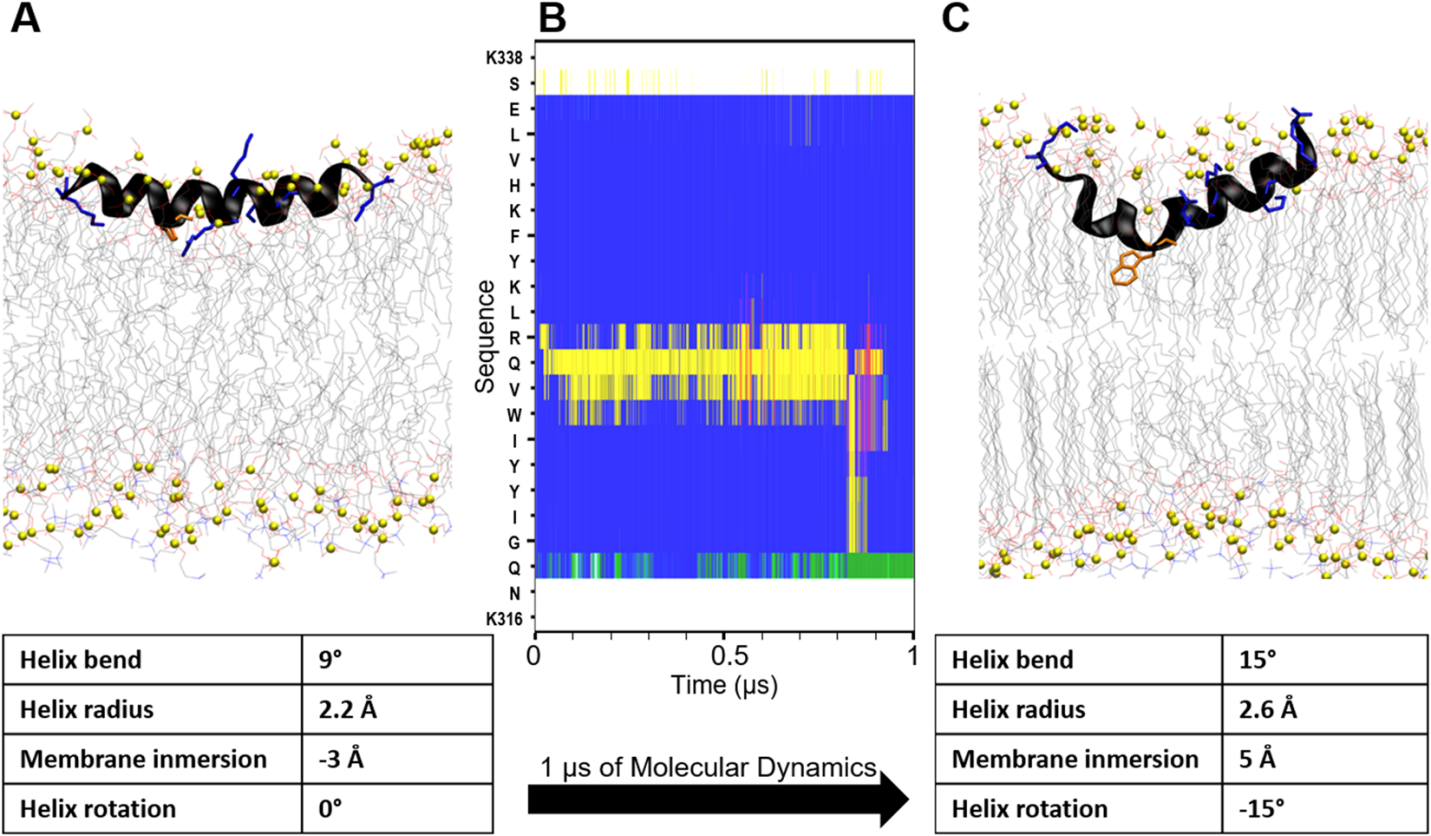
Molecular dynamics simulation of α-helix 4 in the hemimembrane DPPC/DPPM. (**A**) Initial structure. (**B**) Evolution of secondary structure along the sequence (y-axis) during the simulation, computed with DSSP^37^. Blue: α-helix, yellow: turn, green: bend. (**C**) Final structure after 1 µs of MD simulation. Arg and Lys residues of α-helix 4 are represented as blue tubes, Trp residue in orange and the phosphorous atoms of the phospholipids are represented as green spheres. Geometric parameters with relevant changes along the simulation are shown in the tables bellow. Membrane immersion calculated as the distance between the mass centers of peptide and phosphates of the upper hemimembrane.

The helical structures that were initially inserted in the inner hydrophobic space of the membrane (models 1 to 3, with different DPPC:DPPM ratios) did not keep the initial helical fold during the MD simulation and the peptide did not move towards the membrane surface (Fig. S4). In model 4, the helix was placed at the phosphate moieties level of the upper hemimembrane. The helix did not keep the initial helical fold along the full sequence during the MD simulation. The phospholipids that bestride between the membrane and the peptide during the packaging process never moved away along the MD simulation. In model 5, the helix was placed in the aqueous solvent but close to the membrane surface, making interactions between the positive residues of the peptide and the negatively charged atoms of the membrane surface. The major part of the helix unfolded during the MD simulation and never moved to the inner part of the membrane. Only model 6, where the helix was placed at the membrane-water interface of the upper hemimembrane following a soft packing procedure (see methods), kept the α-helix fold during all the MD simulation (Fig. 4). At the beginning of the simulation, the peptide in model 6 changed the orientation of the amino acid side chains towards a clear amphipathic conformation in space: the hydrophobic amino acid residues were oriented towards the inner membrane and the polar amino acid residues were facing the aqueous solvent (Fig. 4C). The helix radius remained quite constant at the end of the MD (2.2Å), with some intervals during the simulation where the helix bending increased the radius (2.6Å) and reversibly unfolded the central part of the helix (Fig. 4B). The helix spontaneously immersed 8Å inside the membrane, but no phospholipids moved on top of the helix so the side-chains of the polar amino acids of the peptide still faced completely the aqueous solvent. The only tryptophan present in the sequence of α-helix 4 (W324) seems to be the main responsible of the hydrophobic interaction with the membrane since it is the most buried residue. This interaction was quite dominant, because the α-helix bended around 6 degrees at this point (Fig. 4C). The helix rotated 15° along its longitudinal axes relative to the initial conformation, looking for a better amphipathic orientation. Electrostatic interactions were stablished between the lateral chains of arginine (R327) and lysines (K316, K329 and K338) of the peptide and the negatively charged oxygens of the phospholipids, but these interactions were not fixed since the membrane is fluid and the phospholipids are moving, with a mean value of 12 polar contacts between the peptide and the membrane along the MD. These characteristics are in agreement with experimental observations of amphipathic helices^28^. In conclusion, α-helix 4 is clearly more stable inserted in a charged membrane model than either free in water or immersed in a fully hydrophobic environment.

### Orientation of amphipathic α-helix 4 in the membrane

**Figure 5:**
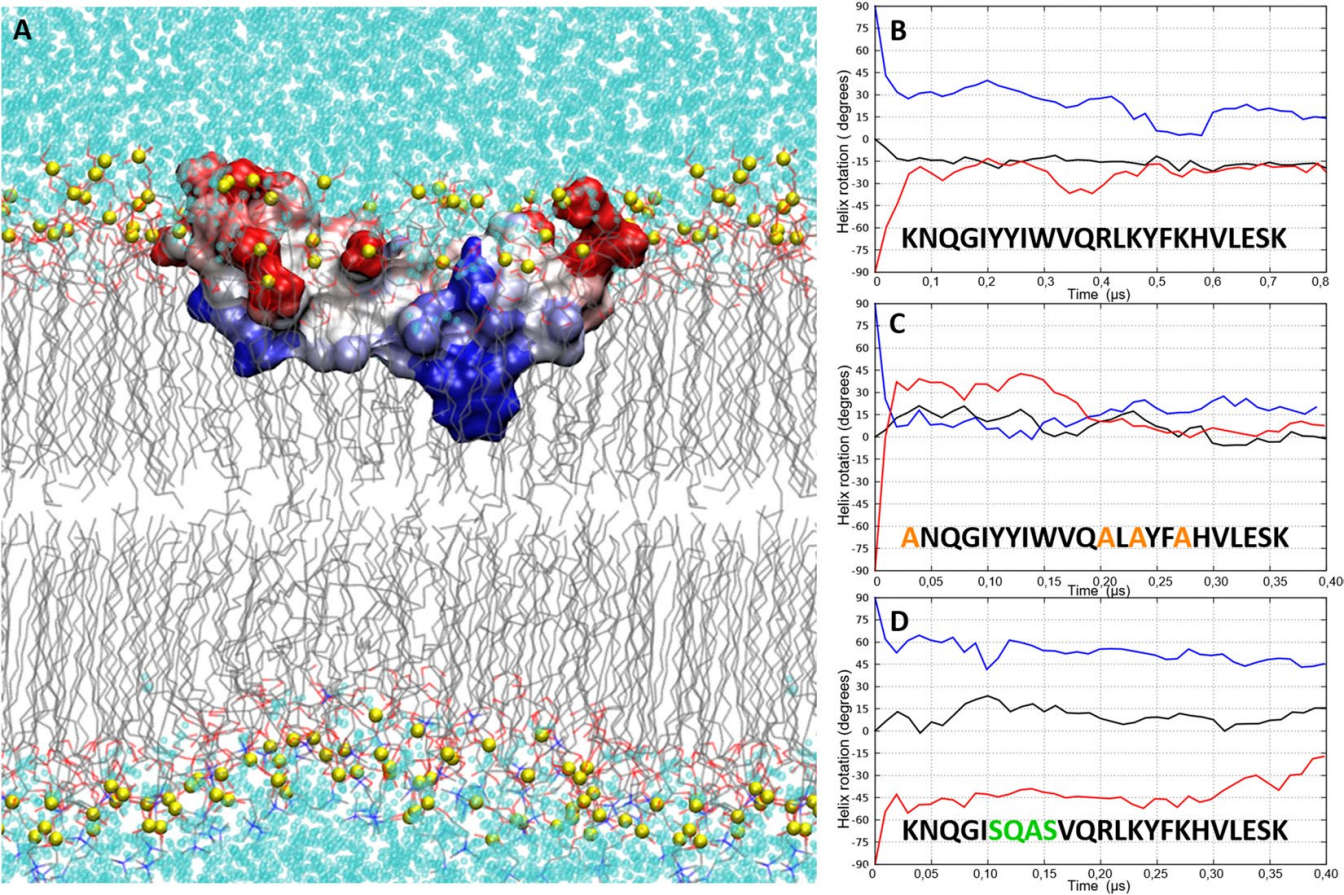
Amphipathic orientation of α-helix 4 of MG517 along the membrane plane. (**A**) Stable geometry of the helix in the DPPC/DPPM hemimembrane. Helix represented as molecular surface colored according to the exposure of hydrophobic (blue) and hydrophilic (red) patches on the peptide surface SAP^46^. DPPC and DPPM lipids represented as thin lines. Phosphorous atoms of the phospholipids are represented as yellow spheres (**B**) Evolution of the amphipathic orientation of α-helix 4 wild type (models 6–8), (**C**) hydrophobic mutant (models 9–11), and (**D**) polar mutant (models 12–14). Black line: initial orientation of Helix 4 as in model 6. Blue line: initial orientation rotated +90 deg. Red line: initial orientation rotated −90 deg.

The orientation of α-helix 4 in the DPPC/DPPM hemimembrane turns out to be an amphipathic orientation in which the hydrophobic surface of the helix is embedded inside the aliphatic chains of the lipids, and at the same time the polar surface is in contact with the charged head groups of the lipids and the solvent (Fig. 5A). The propensity of α-helix 4 to adopt such an amphipathic orientation was tested in the same membrane configuration as in model 6, but with two different initial orientations of the helix. In models 7 and 8 the helix was rotated −90 and +90 degrees, respectively, along its longitudinal axis with respect to the initial orientation in model 6 (Fig. S6). The evolution of the helix rotation was monitored during the MD simulation by measuring the helix orientation with respect to the membrane plane (Fig. 5B). In all three models (6 to 8) and during the first 100 ns of MD, the α-helix 4 rotated back to get the best amphipathic spatial orientation with respect to the membrane and solvent. As described above for model 6, α-helix 4 rotated −15 degrees right at the beginning of the simulation to acquire the most stable amphipathic orientation. In models 7, α-helix 4 rotated back 75 degrees to recover the same amphipathic orientation as in model 6. In model 8, α-helix 4 rotated further 75 degrees, but it could not reach the same final equilibrium orientation as in model 6. This is most likely because the initial electrostatic interactions (reinforced in the starting orientation in model 8) prevented the system to evolve further during the simulation. In any case, irrespective of the initial orientation, α-helix 4 tends to adopt an amphipathic orientation at the membrane interface in which hydrophobic residues are buried in the inner side of the hemimembrane (Fig. 5A).

The role of hydrophobic and polar amino acid residues on the amphipathic orientation of α-helix 4 was tested following the same procedure for two different mutants of α-helix 4. In the first mutant, the helix was made more hydrophobic by substituting the three lysines and the arginine by alanines (**A**_316_NQGIYYIWVQ**A**L**A**YF**A**HVLESK_338_), which causes the deletion of the electrostatic interactions. In the second mutant, the helix was made more polar by substituting the hydrophobic core by polar or less hydrophobic residues, namely Y → S or Q and I → A (K_316_NQGI**SQAS**VQRLKYFKHVLESK_338_). Both mutated helices were embedded in an explicit membrane model (as in model 6) in the initial orientation equivalent to the wild-type helix and rotated −90° and + 90° from its longitudinal axis (models 9–11 for the hydrophobic mutant and models 12–14 for the polar mutant, respectively). All six models were submitted to MD simulations under the same conditions as the wild-type helix described previously. Independently of the initial orientation, the hydrophobic α-helix 4 quickly rotated back to the most stable amphipathic orientation equivalent to that of the wild-type α-helix 4 (Fig. 5C). This hydrophobic mutant was able to rotate almost 120 degrees, indicating that the electrostatics interactions (deleted here) were indeed restricting rotations larger than 75 degrees in the wild-type α-helix 4 (model 8). As this helix remained stable, we conclude that the hydrophobic residues are enough to maintain the helix in the membrane with an amphipathic orientation. On the other hand, the polar mutant of α-helix 4 could not rotate more than 45 degrees and was unable to reach the stable wild-type orientation (Fig. 5D). Therefore, we conclude that the hydrophobic core is indeed the main responsible for the amphipathic orientation of the peptide, placing W324 in the inner part of the hemimembrane as reflected in models 6, 7 and 8.

### Truncation of the amphipathic α-helix 4

**Figure 6:**
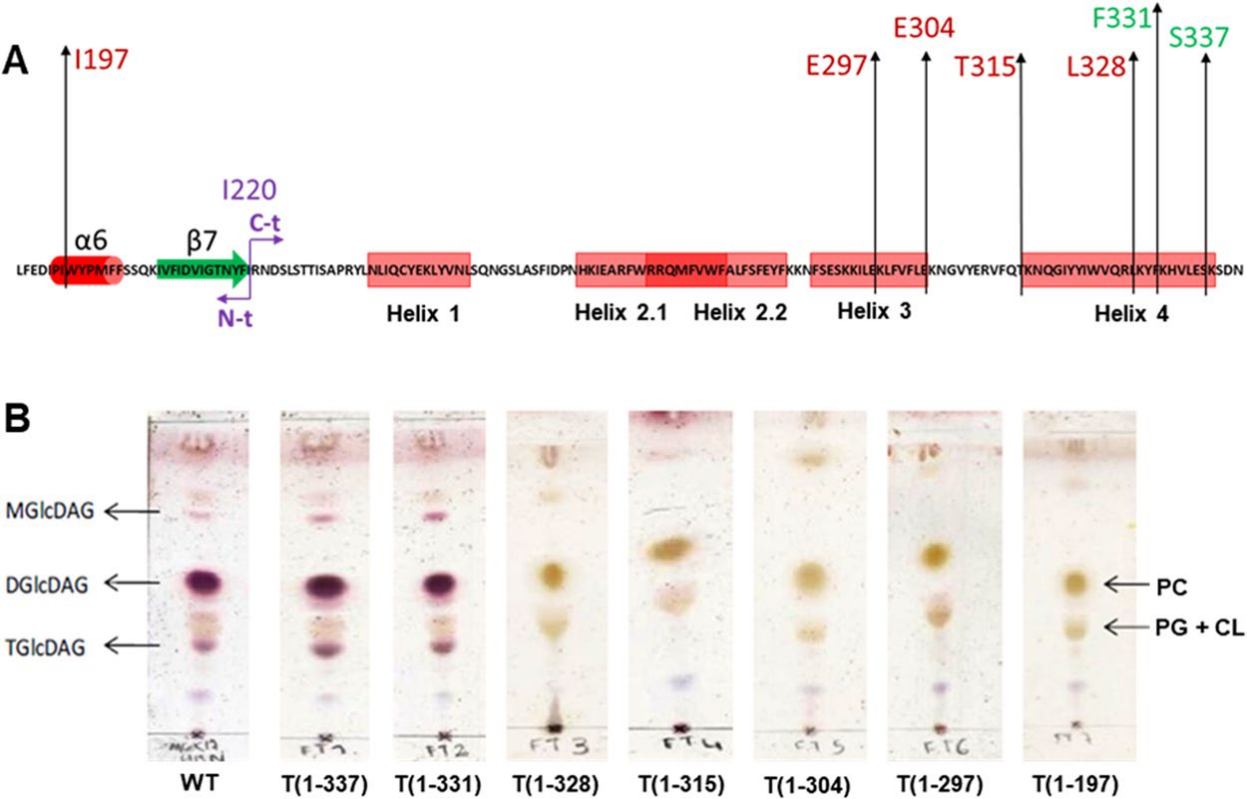
Glycolipids production of GT MG517 and C-terminus truncated forms. (**A**) C-terminus sequence indicating the last aminoacid of each truncated form. (**B**) TLC analysis of lipid extracts from recombinant *E. coli* cells expressing the wild type and truncated proteins. MGlcDAG, DGlcDAG and TGlcDAG: mono- di, and tri-glucosyldiacylglycerol, respectively; PE, phosphatidylethanolamine; PG: phosphatidyl-glycerol; CL: cardiolipine.

To evaluate the role of the C-terminus α-helix 4 on enzyme activity, a series of truncated proteins were designed and recombinantly expressed. Truncations were defined at each of the six last lysine residues of the sequence, T(1–337), T(1–331), T(1–328), T(1–315), T(1–304), T(1–297), and a full truncation of the C-terminus region T(1–197) (Fig. 6A). The proteins were expressed and purified as reported for the wild type (wt) enzyme^18^ yielding soluble proteins. Activity was evaluated on cell extracts monitoring glycoglycerolipid products formation (see Methods). Whereas truncation at positions 337 and 331 did yield active enzymes, the activity was lost at the truncated protein T(1–328), which corresponds to the deletion of the 13 last C-terminus amino acids (K329 to N341) (Fig. 6B). This truncated form represents the deletion of the last 10 amino acid residues of the amphipathic α-helix 4.

We modelled the corresponding truncated helix (K316-L328) and performed MD simulations (model 15) with the same previous conditions of the folded peptide in a membrane (as in model 6). This shorter helix exhibited a similar behavior as the whole α-helix 4, remaining attached to the hemimembrane with an amphipathic arrangement of its residues (Fig. S5). This truncated form still preserves W324 and K316. However, the loss of 3 interactions with the membrane (R327, K329 and K338), out of the 5 possible in the whole helix, necessarily reduces the stability of this truncated helix in the membrane. A weaker interaction of GT MG517 truncated at this position with the membrane may explain the loss of enzymatic activity, albeit the truncation does not affect MG517 protein stability and solubility.

### Interaction energy of C-terminus amphipathic α-helix 4 with the membrane

**Figure 7:**
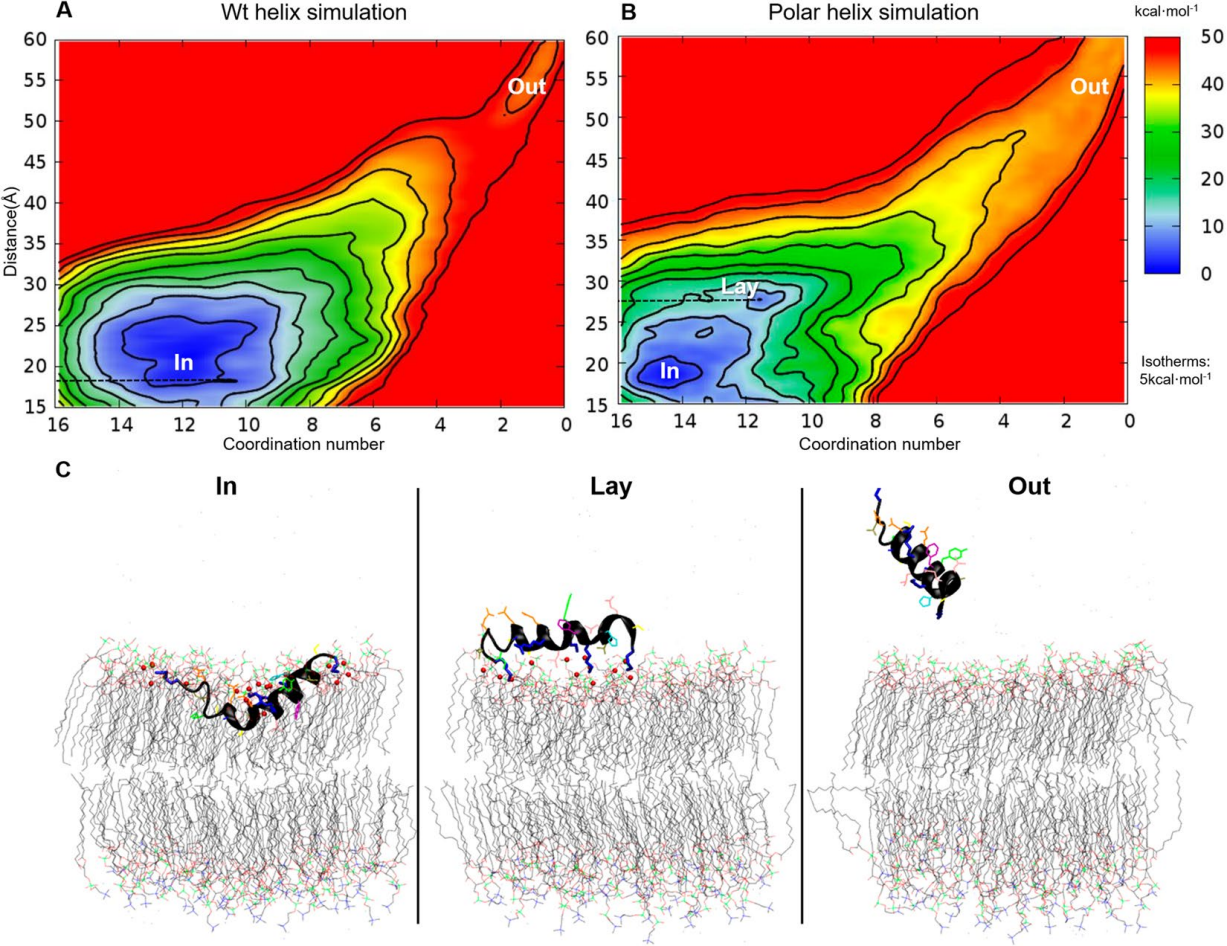
Free energy landscape of GT MG517 α-helix 4 binding to DPPC/DPPM hemimembrane as a function of the distance between the centers of masses (y-axes) and contacts between polar sidechains of the helix and charged head groups of the lipids (x-axes). (**A**) Wild type and (**B**) Polar mutant of the helix. Isotherms levels, shown as black lines, are located at 5 kcal/mol levels. Three main states are labelled: “In”, helix inserted in the membrane; “Out”, helix free in the solvent, detached from the membrane; and “Lay”, helix lying on top of the membrane fully exposed to the solvent. (**C**) Representative structures of the three main states.

GT MG517 α-helix 4 is an amphipathic helix, stable in a DPPC/DPPM membrane by means of a peripheral monotopic interaction involving the positively charged residues of the peptide and membrane phospholipids, and the essential hydrophobic residues of the peptide and the inner membrane. The formation of the complex in the vicinity of the membrane was further characterized with Metadynamics (MetaD) calculations. The unbinding of α-helix 4 from the lipid membrane was simulated for both the wild type and the polar mutant as a function of two reaction coordinates, or collective variables (CV): i) the coordination number between positively charged groups of the helices and negative groups of the membrane (CV1), and ii) the distance between the centers of masses of the helix and the membrane (CV2) (Fig. S7). During the MetaD simulation, the peptides were fully extracted from the membrane to the solvent and forced to completely enter from the solvent to the inner membrane. An estimation of the energy landscape associated to the early stages of this process was reconstructed from the MetaD simulation data following standardized procedures (see methods) (Fig. 7). The most stable position of both the wt and polar mutant of α-helix 4 was the same found in the MD at a CV2 distance of 20Å between the centers of masses of the helix and the membrane (“In” state in Fig. 7). The number of polar interactions between both the wt and polar mutant of the peptide (CV1) in this stable position was also the same as identified previously by MD (12 and 15 polar interactions respectively). Notably, the association between the polar helix and the membrane reveals the existence of an additional metastable state located at a CV2 distance of 26Å, in which the peptide is placed on the external surface of the membrane (“Lay” state in Fig. 7). Albeit lacking the hydrophobic core, the polar helix can remain associated to the outer part of the membrane by means of just electrostatics interactions. There is a subtle energetic barrier for the “Lay” to “In” transition for the polar helix. On the contrary, both states are indistinguishable in terms of energy for the wild type helix. This indicates that the hydrophobic core present in wild type α-helix 4, not only contributes to the helix orientation, but it favors the immersion of the helix into the inner part of the membrane as well. For both simulations an additional region is explored (“Out” in Fig. 7) in which all contacts (CV1) between the helix and the membrane have been lost. Extraction of the polar mutant helix from the “In” state to the “Out” state requires less energy than the wild-type during the simulation. Combining this data with the previous MD simulations, it can be concluded that the insertion of the MG517 C-terminus α-helix 4 into the membrane proceeds smoothly from the external surface to the inner part of the membrane, and that the helix adopts an amphipathic orientation once it reaches the surface of the membrane, resulting in a tight helix-membrane binding.

## Conclusion

The biosynthesis of membrane glycolipids in *Mycoplasma genitalium* takes place via a single and essential gene that encodes for the MG517 glycosyltransferase enzyme. Despite requiring the presence of phospholipid micelles for enzymatic activity, no clear indications of membrane association sequence patterns can be identified along the sequence of MG517. The prediction of transmembrane domains, hydropathy profiles, and amphipathic helical wheel analysis are not conclusive *per se*. This seems to be a shared difficulty for peripheral membrane proteins with a weak monotopic association to the cell-membrane in general. We have lined up here a combination of standard computational techniques that allow identifying and characterizing amphipathic helices that may be involved in the membrane association process of peripheral membrane proteins. By combining sequence analysis tools, molecular dynamics simulations in explicit membranes of different composition and explicit solvent, and metadynamics, we have found evidences for the existence of an amphipathic helix located in the C-terminus apical part of GT MG517 (316–338 aa). It binds to DPPC/DPPM membranes by a monotopic adhesion, embedding the hydrophobic amino acids of the helix in the hydrophobic core of the membrane, and the polar ones faced to the charged head-groups of the lipids. This amphipathic character of the helix has been verified by *in-silico* mutagenesis of the hydrophobic amino acids, resulting in a dissociation of the helix from the inner part of the membrane. Not only this helix is essential for membrane association, but it is also essential for enzymatic activity as shown by the drastic decrease in glycolipids production of truncated forms of MG517 at different positions along this helix.

Based on the results here reported on the nature of the C-terminus region of MG517, we propose that the C-terminus helix 4 is the main structural element guiding the association of the protein to the membrane for proper substrate binding. By combining the previously generated model for the N-terminus region of GT-MG517^17^ with the new data obtained here for the C-terminus region, we speculate a tentative model for the full-length protein association to the membrane as summarized in the Supplemental Information. Nevertheless, new attempts to crystallize the protein to solve the 3D structure deserve special attention to unravel the details of such peripheral protein-membrane association.

## Methods

### Sequence analysis, modeling and molecular dynamics of α-helices

The amino acid sequence of *Mycoplasma genitalium* MG517 (Uniprot accession code: Q9ZB73) was analyzed with PsiPred version 3.2^29^ and MPEx Version 3.2.11^24^ using default parameters values. Only PsiPred scores higher than 6 were considered. The membrane interaction prediction from MPEx, combined with the PsiPred results was used to select 5 putative membrane associated helices: 1, 2, 2.1, 3 and 4 (Fig. 1). The sequences of the 8 helices (the five predicted plus 3 mutants of α-helix 4) considered in this work are: Helix 1: LIQCYEKLYVNLS, Helix 2: KIEARFWRRQM FVWFA, Helix 2.1: RRQMFVWFALFSFEYFKK, Helix 3 FSESKKILEKLFVFLE, α-helix 4 (wild type) KNQGIYYIWVQRLKYFKHVLESK, α-helix 4 (hydrophobic mutant) ANQGIYYI WVQALAYFAHVLESK, α-helix 4 (polar mutant) KNQGISQASVQRLKYFKHVLESK, α-helix 4 (truncated) KNQGISQASVQRL. The initial structure of these helices was obtained with MODELLER v.9.8^30^, in which all amino acids were constrained to an α-helix secondary structure.

Unconstrained Molecular Dynamics (MD) simulations were performed for each helix with GROMACS v4.5.3^31^. The simulations were performed with the amber03 force-field^17^ and explicit solvent treatment with the tip3p force-field^32^ in a cubic box of 3 nm side under periodic boundary conditions. Counter-ions (Na^+^ and Cl^−^) were added to neutralize the total charge of the system at a final concentration of 0.15 M. Initial coordinates of each system were previously equilibrated by means of five runs of conjugated gradient energy minimization (1000 steps each run) followed by 50000 steps of MD at 300 K with the helix coordinates fixed. The target simulation temperature (300 K) was achieved after 10^5^ MD steps starting at 175 K in which the temperature was gradually increased in 6 steps up to 300 K by coupling the system to a Nosé-Hoover thermostat^33^. Equilibration was completed with 10^5^ MD steps in which the size of the simulation box was adapted with a Parrinello-Rahman barostat^34^ to reach a final equilibrium pressure of 1 bar. All simulations were performed with all bonds constrained by means of the LINCS algorithm^35^, and a simulation time step of 2 fs. Fast Particle-Mesh (PME)^36^ was applied to treat long-range non-bonding interactions. Explicit van der Waals and Coulomb potentials were used up to a cut-off distance of 0.8 nm. All simulations were performed under the NPT ensemble at 300 K and 1 bar by coupling to the Nosé-Hoover thermostat and Parrinello-Rahman barostat. Total simulation time for each simulated helix was: 4 µs for Helix 1, 2 µs for Helices 2, 2.1 and 4, 1.8 µs for Helix 3.

The secondary structure evolution along the simulations was monitored with the GROMACS package tool *do_dssp*, using the DSSP software^37^. Resulting structures from each simulation were grouped into 100–200 structures using the *gromos* clustering method^38^ with a cut-off value of 0.3 nm. The middle structure of the most populated group was chosen as representative of each helix for further analysis and graphical representations.

### Molecular dynamics of α-helix 4 in a membrane environment

A structural model of an equilibrated bi-layer lipid membrane composed of 128 dipalmitoyl phosphatidylcholine (DPPC) molecules was obtained^39^. Membrane composition was modified to generate different initial membrane models with varying DPPC and dipalmitoyl phosphatidylmethane (DPPM) ratios by manually replacing the choline by a methyl group in the structure of selected DPPC molecules in the original membrane model. The representative structures of α-helix 4 (wild type, polar and hydrophobic mutants and truncated) from the simulations in solution were separately inserted in these membrane models following the membrane inflating and shrinking protocol^40^. A final total area per lipid of 70 Å^2^ was obtained. Different initial positions and rotations of the helix with respect to the membrane were generated with the GROMACS package tool *editconv*, being the principal axes of the helix always parallel to polar head groups of the membrane. In total, 15 different initial structures of different α-helix 4 variants and positions embedded in different DPPC/DPPM membrane compositions were generated: Model 1: upper hemimembrane: 64DPPC + lower hemimembrane: 64 DPPC + α-helix 4 wild type placed in the center of the bilayer, Model 2: upper hemimembrane: 64DPPC + lower hemimembrane: 64 DPPM + α-helix 4 wild type placed in the center of the bilayer, Model 3: upper hemimembrane: 64DPPC + lower hemimembrane: 37 DPPM:31DPPM + α-helix 4 wild type placed in the center of the bilayer, Model 4: upper hemimembrane: 64DPPM + lower hemimembrane: 64 DPPC + α-helix 4 wild type placed adjacent to phosphate groups of DPPM, allowing DPPM molecules to cover it. Model 5: upper hemimembrane: 64DPPM + lower hemimembrane: 64 DPPC + α-helix 4 wild type placed on top of the DPPM layer exposed to the solvent, with a 45 degrees rotation along the principal axes of the helix to allow polar contacts between the helix and the phosphate groups of DPPM. Model 6: upper hemimembrane: 64DPPC + lower hemimembrane: 64 DPPM + α-helix 4 wild type placed adjacent to phosphate groups of DPPM, not allowing DPPM molecules to cover it (this is accomplished with a gentle shrinking in the lipids packaging procedure). Models 7 and 8 are equivalent to model 6 but with a −90 and 90 degrees rotation along the principal axes of the helix respectively. Models 9, 10 and 11 are equivalent to models 6, 7 and 8, respectively, but with the hydrophobic mutant of α-helix 4. Models 12, 13 and 14 are equivalent to models 6, 7 and 8, respectively, but with the polar mutant of α-helix 4. Finally, Model 15 is equivalent to model 6 but with the truncated form of α-helix 4.

Models 1 to 15 were embedded in explicit solvent. Molecular dynamics simulations of the resulting systems 1 to 15 were performed in equivalent conditions and following the same protocol as described above but setting the temperature, in this case, to 323 K that is above the phase transition temperature of the lipid^40^. The force-field was switched to gromos53a6^41^ combined with Berger^42^ for the helix and lipids description respectively. The initial size of the simulation box was 6.4 × 6.4 × 6.6 nm in all systems. All simulations involving protein-lipid interactions were treated with the same force-field scheme and simulation conditions. The coordinate’s evolution of all molecular dynamic simulations were visualized and analyzed with VMD^43^.

### Metadynamics of α-helix 4 binding to the membrane. Interaction energies

Model 6 was used as a starting configuration to simulate the association of α-helix 4 to the DPPC/DPPM membrane. Two collective variables were used to describe this process under the metadynamics scheme: i) number of contacts between the N atoms of residues K316, R327, K329, K332 and K337 with all oxygens of the DPPM hemimembrane (CV1), (ii) distance between the center of masses of the peptide and the membrane (CV2). A switch function (equation S1 in Supplementary Information) with parameters n = 5, m = 6, d_0_ = 0.4 nm, and r_0_ = 0.04 nm was used for CV1 as implemented in PLUMED v1.3^44^. The exploration of CV2 was limited to the range 1.5 nm to 6.0 nm by the use of parabolic walls (force-constant = 10^6^ kJ/(mol·nm^2^), exponent = 2). The width of the gaussian terms used for the metadynamics history-dependent potential were set to the range 0.025 nm to 0.05 nm for CV1, and between 2 and 8 for CV2. The height of the gaussians were set to 4 kJ/mol. The secondary structure of the helix along the whole simulation was kept fixed by an additional restraint. Hills deposition time was constant at 20 ps. Total simulation time was extended to 534.5 ns and 217.5 ns for the wild type and polar mutant simulations respectively. Metadynamics simulation data and the resulting free energy maps were reconstructed with METAGUI plugin^45^. Assessment on the convergence of the Metadynamics simulations is provided in Fig. S8.

### Expression of C-terminus truncated forms of GT MG517

The plasmid pET44b(+)−*mg517*^18^ was used for the construction of the different C-terminus tructated forms of GT MG517. The coding sequence was amplified by PCR with Pfu Turbo (Stratagene) using one forward primer with a *Nde*I restriction site and a reverse primer for each truncated form with a *Bam*H restriction site (Table S1). The amplified fragments were digested with *Nde*I/*Bam*H and ligated (T4 DNA ligase) to a pET15b(+) vector previously digested with the same restriction enzymes. The new constructs contained a HisTag at the N-terminus of the expressed proteins for affinity chromatography purification. All constructs were verified by DNA sequencing.

The truncated GT MG517 proteins (T(1–337), T(1–331), T(1–328), T(1–315), T(1–304), T(1–297), and T(1–197)) and the full length protein (1–341) were expressed as reported for the wt enzyme^18^. Briefly, *E. coli* BL21(DE3Star) cells were co-transformed with pET15b-mg517-T (wt and truncated forms), and pGro7 (from Takara Bio Ltd), a plasmid encoding for *E. coli* chaperones GroEL and GroES under the control of an araB promoter and containing a chloramphenicol-resistance gene. Cells were grown in LB medium containing ampicillin (100 μg/mL) and chloramphenicol (25 μg/mL) at 37 °C. When the optical density of the culture reached 0.3, expression of chaperones was induced by adding L-arabinose (2 g/L). After 30 min at 37 °C, IPTG (1 mM) was added and cells grown for 16 h at 25 °C. Cell were harvested by centrifugation at 5000 g.

All truncated form were extracted and purified as soluble proteins following the protocol reported for the wt enzyme (18). They were purified to homogeneity as judged by SDS-PAGE, and MALDI-TOF mass spectrometry analysis confirmed the identity of each truncated form.

### Enzyme activity of GT MG517 wt and truncated forms

Since *E. coli* does not produce endogenous glycoglycerolipds, the activity of recombinantly expressed GT MG517 and its truncated forms can be directly detected *in vivo* by analyzing the lipidic composition of the cell extract^17^. The cellular pellet of *E. coli* BL21(DE3) cell expressing the wt and truncated forms was washed with 0.9% NaCl solution and subjected to lipids extraction with chloroform/methanol 2:1 (v/v) in a ultrasounds water bath. The organic fraction was concentrated by solvent evaporation under a steam of nitrogen and analyzed by TLC (silica gel plates) developed with chloroform/methanol/water 65:35:4 (v/v) and stained with sulfuric/methanol/water (45:45:10 v/v) for visualization. Glycoglycerolipids stain first as purple spots whereas phospholipids appear as pale brown spots.

## Supplementary information

Supplementary information
