# Supplementary material for: Essential Mycoplasma Glycolipid Synthase Adheres to the Cell Membrane by Means of an Amphipathic Helix
Source: Sci Rep. 2019 May 8;9:7085. doi: 10.1038/s41598-019-42970-9 (PMC6506492; doi:10.1038/s41598-019-42970-9)
Supplement: Supplementary file 1 — Supplementary information [file 41598_2019_42970_MOESM1_ESM.pdf]

Supplementary Information

ESSENTIAL MYCOPLASMA GLYCOLIPID SYNTHASE ADHERES TO THE CELL MEMBRANE BY MEANS OF AN AMPHIPATHIC HELIX

J. Romero-García, X. Biarnés*, A. Planas*

Laboratory of Biochemistry, Institut Químic de Sarrià, Universitat Ramon Llull, Barcelona, Spain

Table S1. Primers used for construction of the C-terminus truncated forms of GT MG517.

Figure S1. Structures generated by Robetta[1] server for the C-terminus sequence of GT MG517.

Figure S2. Secondary structure prediction for MG517 protein from PSIPRED server.

Figure S3. Molecular dynamics simulations of Helix 1, 2.1, 2.1, and 3 in aqueous solvent.

Figure S4. Molecular dynamics simulations of solvent-membrane systems 1 to 5.

Figure S5. Truncated helix $K_{316}NQGIYYIWVQRL_{328}$.

Figure S6. Helix rotation simulations.

Figure S7. CVs used in the MetaDynamics simulations: “Distance” and “Coordination”.

Equation S1. Switch function used for the collective variable CV1

Figure S8. Convergence of Metadynamics simulations.

Tentative structural model for GT MG517 interaction with the membrane

Figure S9. Modeled structure for the N-terminus region of GT MG517 reported in ref 2.

Figure S10. Hydropathic profile of the full-length MG517 protein.

Figure S11. Proposed overall topology of full-length MG517.

Table S1. Primers used for construction of the C-terminus truncated forms of GT MG517.

Forward	5’-TGGGAATTCCATATGGATAAACTTGTTAGTATATT-3’
Reverse T(1-337)	5’-GTCGGGGATCCTTAAGATTCCAAAACATG-3’
Reverse T(1-331)	5’-GTCGGGGATCCTTAAAAATATTTTAGTCGCT-3’
Reverse T(1-328)	5’-GTCGGGGATCCTTATAGTCGCTGTACCCAAA-3’
Reverse T(1-315)	5’-GTCGGGGATCCTTATGTTTGAAAAACACGT-3’
Reverse T(1-304)	5’-GTCGGGGATCCTTATTCCAAAAAAACAAATAG-3’
Reverse T(1-297)	5’-GTCGGGGATCCTTATTCAAGAATTTTTTTTG-3’
Reverse T(1-197)	5’-GTCGGGGATCCTTAGATTGGTATATCTTCAAA-3’

Figure S1. Structures generated by Robetta server for the C-terminus sequence of GT MG517 (residues 222 to 341). Colored from N-terminus (red) to C-terminus (blue).

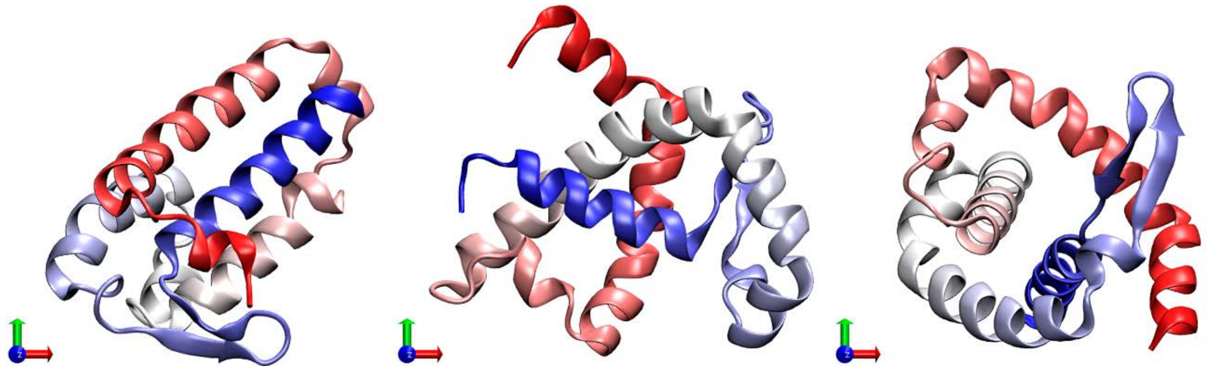

Figure S2. Secondary structure prediction for MG517 protein from the PSIPRED server. The C-terminus region starts at residue 222 (black arrow).

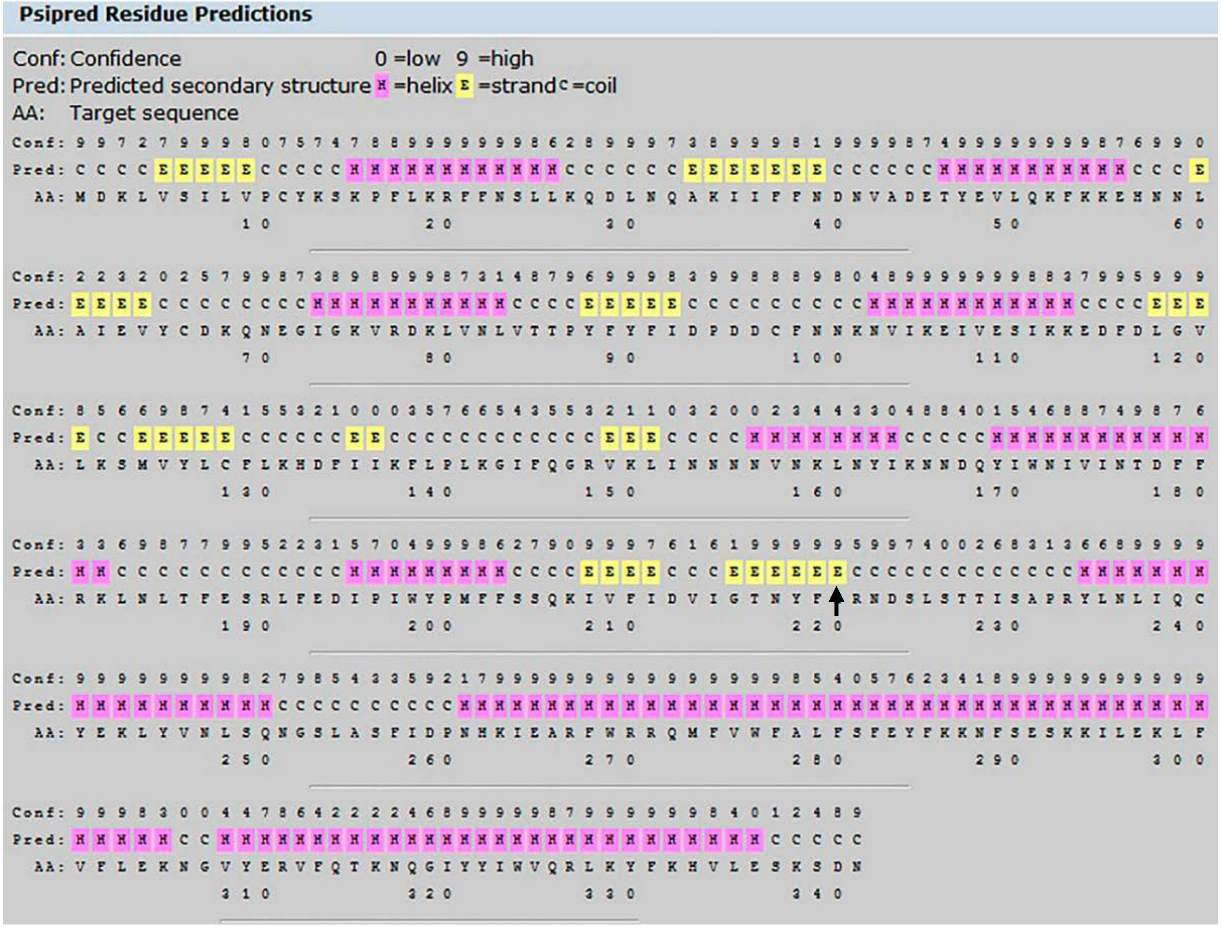

Figure S3. Molecular dynamics simulations of Helix 1, 2, 2.1, and 3 in aqueous solvent. Secondary structure evolution (computed with DSSP). Blue: α-helix, yellow: turn, green: bend.

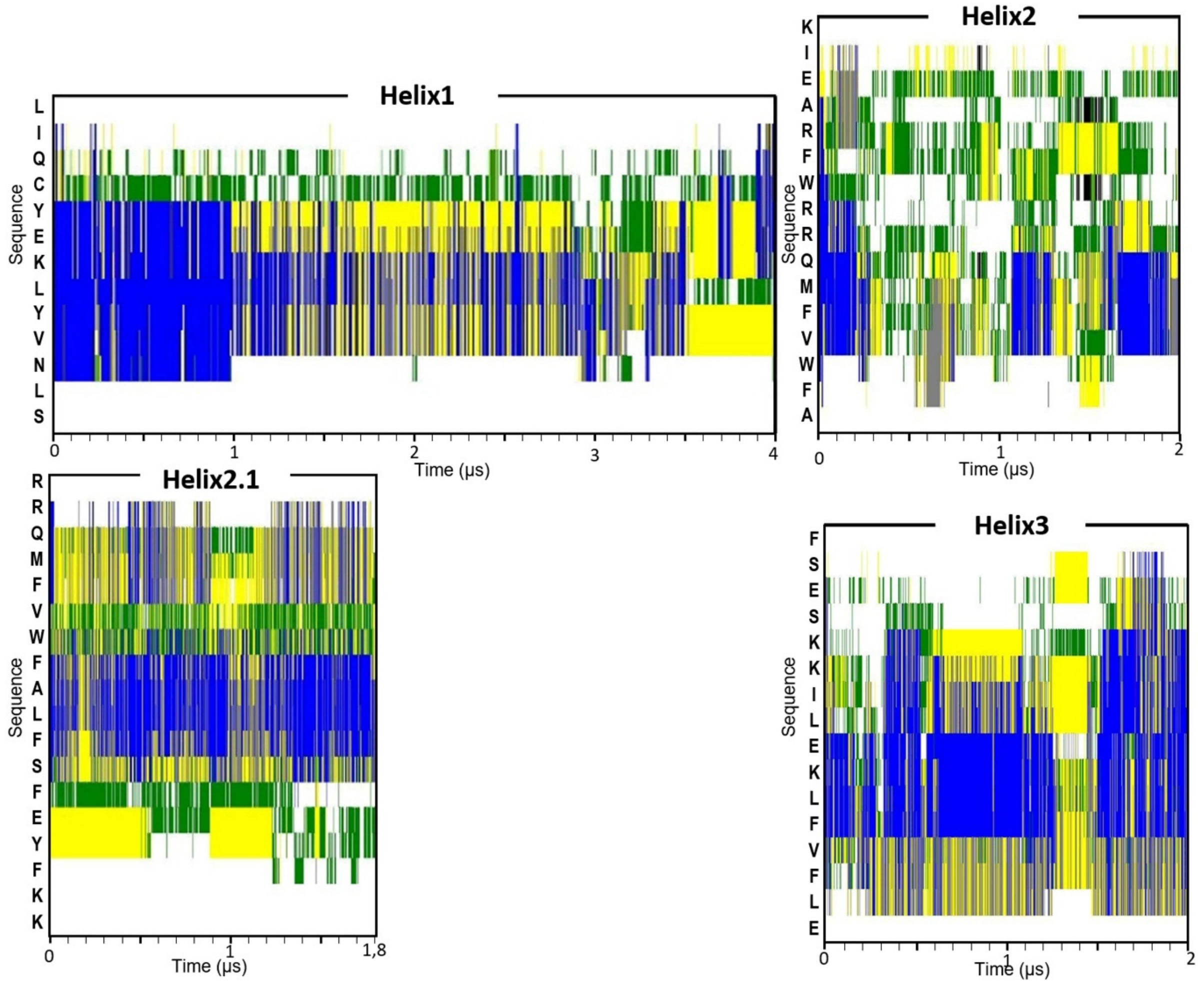

Figure S4. Molecular dynamics simulations of solvent-membrane models. A) Models 1-5 from Figure 3, initial structures and after 1 ns MD. The green and blue spheres represent, respectively, the phosphorous atoms of the phospholipids (present in both DPPC and DPPM) and the nitrogen atoms of the phosphatidylcholine lipids (present only in DPPC). B) Secondary structure evolution (computed with DSSP) along MD trajectories for models 1 to 5. Blue: α-helix, yellow: turn, green: bend, red: β-sheet, purple: 5-helix.

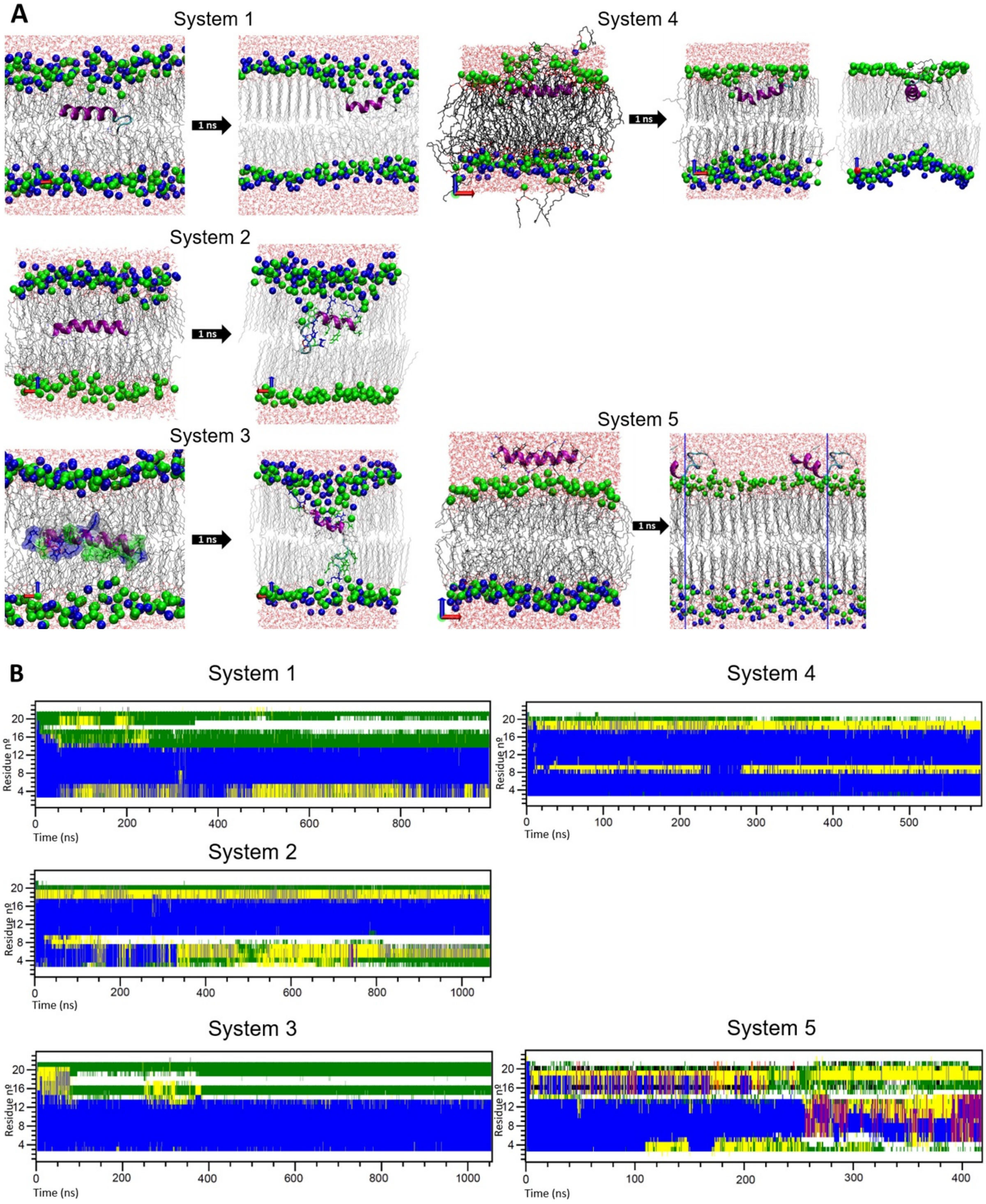

Figure S5. Truncated helix $K_{316}NQGIYYIWVQRL_{328}$. The peptide is stable in the membrane, with an amphipathic arrangement of the amino acid residue side chains. Green and blue: polar residues. White: hydrophobic residues.

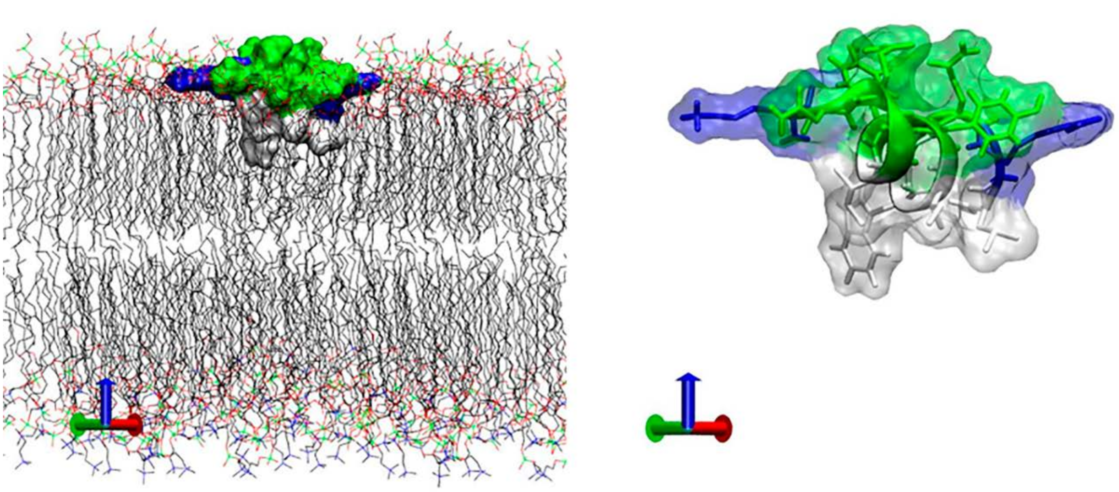

Figure S6. Helix rotation simulations. A) Helix 4 initial orientation in model 6. The helical structure comes from the most representative extended conformation of the Helix 4 MD (free in water). B) The system is built with a 90° rotated helix 4 from the initial position (A). C) The system is built with a -90° rotated helix 4 from the initial position (a).

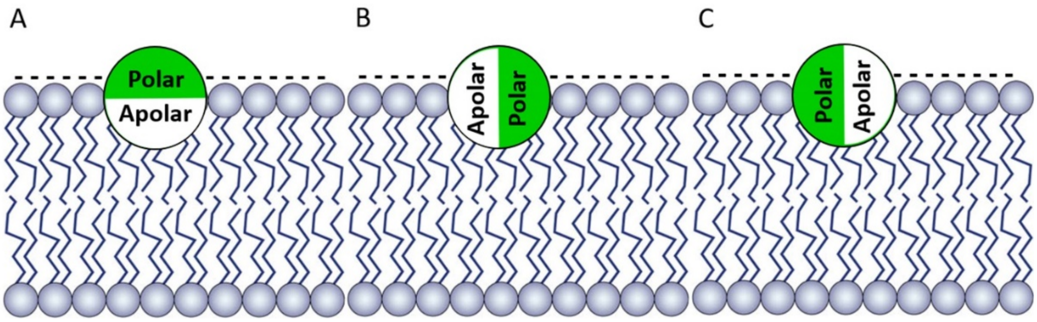

Figure S7. Collective variables (CV) used in the Metadynamic simulations: "Distance" (CV1) and "Coordination number" (CV2). All the lipid phosphate oxygens (red spheres) closer than 0.3 Å from the side chain nitrogens of Arg and Lys peptide residues are showed in the picture.

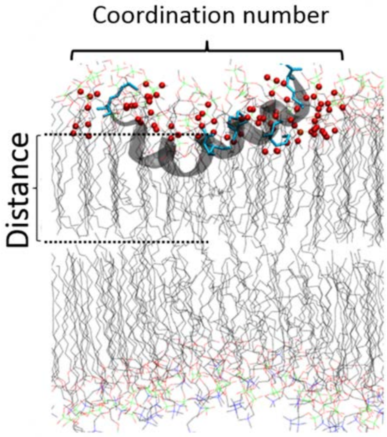

Equation S1. Switch function used for the collective variable CV1, with parameters n=5, m=6, d_0=0.4, and r_0=0.04 as implemented in PLUMED v1.3[45].

$$S_{ij} = \begin{cases} 1 & \text{for } r_{ij} \leq 0 \\ \frac{1-\left(\frac{r_{ij}}{r_0}\right)^n}{1-\left(\frac{r_{ij}}{r_0}\right)^m} & \text{for } r_{ij} > 0 \end{cases} \qquad \textit{where } r_{ij} = |r_i - r_j| - d_0$$

Figure S8. Convergence of Metadynamcis simulations. The evolution of the bias potentials projected on CV2 (coordination number) is computed at different simulation times and averaged over the last 1000 added hills. A) Wild-type helix-4 simulation. Even-growth of the bias potential is observed between 9000 and 27000 hills (180ns to 540ns) in the whole range of CV2 values. B) Polar mutant helix-4 simulation. Even-growth of the bias potential is observed between 7000 and 22000 hills (70ns to 220ns) in the CV2 range from 12 to 17.

A

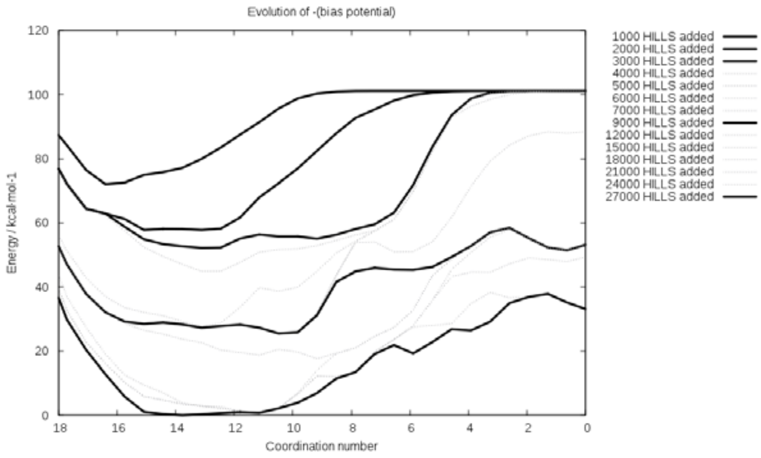

B

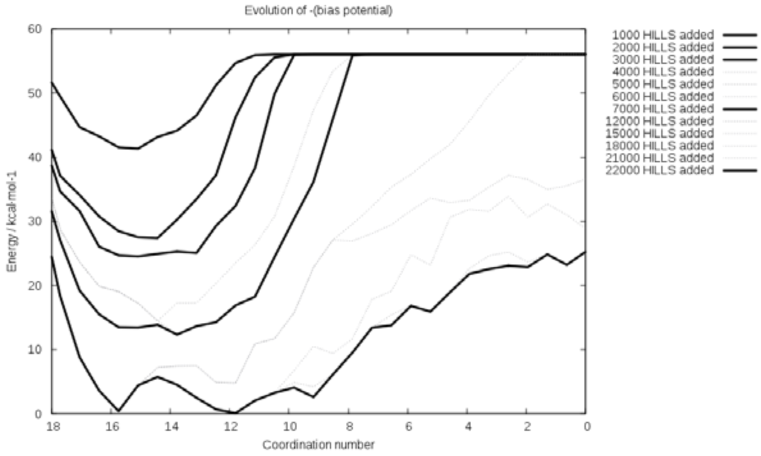

Tentative structural model for GT MG517 interaction with the membrane

In addition to α-helix 4, other hydrophobic α-helices along the C-terminus region were identified (Figure 1). We can now complement the structural information we previously generated for the N-terminus region of GT-MG517[2] with the new data obtained here for the C-terminus region. The modeled N-terminus region (aa 1 to 220) is characterized by a GTA fold comprising six α-helices and seven β-strands (Figure S9). Between β-strands 5 and 6, a variable region, with no significant sequence similarity among GT2 enzymes, was proposed to be a flexible element involved in acceptor binding. This variable region contains an initial short α-helix that has a hydropathic profile and was stable after long MD simulations, but the rest of the variable region could not be resolved in the different modelled structures. In an attempt to combine the reported model for the N-terminus region[2] and the helixes here predicted for the C-terminus region, we propose a hypothetical topological model for MG517 as presented in Figure S11. The first C-terminus α-helix (Helix 1) could cover the hydrophobic patch observed in the models for the N-terminus region. Helix 2 is highly hydrophobic and, not being proposed as interacting with the membrane by any of the predictors used, is likely to be buried and interact with other protein elements. The lower hydrophobicity of Helix 3 (the lowest among the 5 helices at the C-terminus), suggests that it may be partially solvent-exposed. The C-terminus region could be allocated below the N-terminus region close to the *variable region* that was proposed to be involved in DAG acceptor substrate binding[2]. This variable region (amino acids 126 to 169) contains a small α-helix that has a hydropathic profile compatible with membrane association (Figure S10). Given the fact that the DAG acceptor molecule is embedded in the membrane and the *variable region* interacts with the acceptor, both helices, the amphipathic C-terminus α-helix 4 and the helix belonging to the *variable region*, could be the elements that are responsible for the peripheral association of GT MG517 to the membrane, making a global picture as depicted in Figure S11. MG517 is a sequentially-acting enzyme able to transfer a glucosyl unit to different substrates of unalike polarity (either DAG or GlcDAG acceptors)[3]. The enzyme activity seems to be modulated by the cell membrane curvature which regulates the synthesis of non-bilayer-prone and bilayer-forming glucolipids[4]. The dual activity of MG517 necessarily requires a geometrical rearrangement of the active site to accommodate acceptors of different nature. For this reason, we speculate for the necessary existence of an additional element connecting the amphipathic helix membrane anchor with the catalytic protein core which would allow changing the shape and hydrophobic properties of the active site to allow for the accommodation of different acceptors. We hypothesize that some hydrophobic loop adjacent to the amphipathic α-helix 4 could be the elements that transfers the membrane curvature information, from the C-terminus amphipathic α-helix 4 to the *variable region*, which may modulate the acceptor specificity for DAG or GlcDAG substrates. This topology is compatible with the Robetta modeled

structure for the C-terminus region of MG517 (Figure S1, central structure). This model is composed of five α-helices where the amphipathic α-helix 4 has an orientation able to interaction with the membrane. The N-t helix (helix 1) is placed at the top and may interact with helix α6 of the modeled N-terminus region (Figure S11). The internal helix 2, which show the most hydrophobic moment (Figure 1), could be buried and covered by helix 1 and helix 3. The sequence between helix 3 and the amphipathic α-helix 4, predicted as a β-sheet by Robetta, may get close to the N-terminus *variable region* and be the structural element that transfers the membrane physicochemical properties from the amphipathic helix interacting with the membrane to the sugar acceptor environment involving the variable region. Although highly speculative, this tentative model may guide mutagenesis studies addressed to probe the interactions between the different structural elements and the membrane to evaluate enzyme activity and regulation.

Figure S9. Modeled structure for the N-terminus region of GT MG517 reported in Romero-García *et al.*[2]. A) Consensus topology proposed for GTA proteins. B) MG51 N-terminus region model based on 2FFU PDB structure. C) MG517 model after 1 μs of Molecular Dynamics. The variable region is colored in steel blue and surrounded by a dotted line in B and C. The arrows mark the region that rearranges to an α-helix during the simulation.

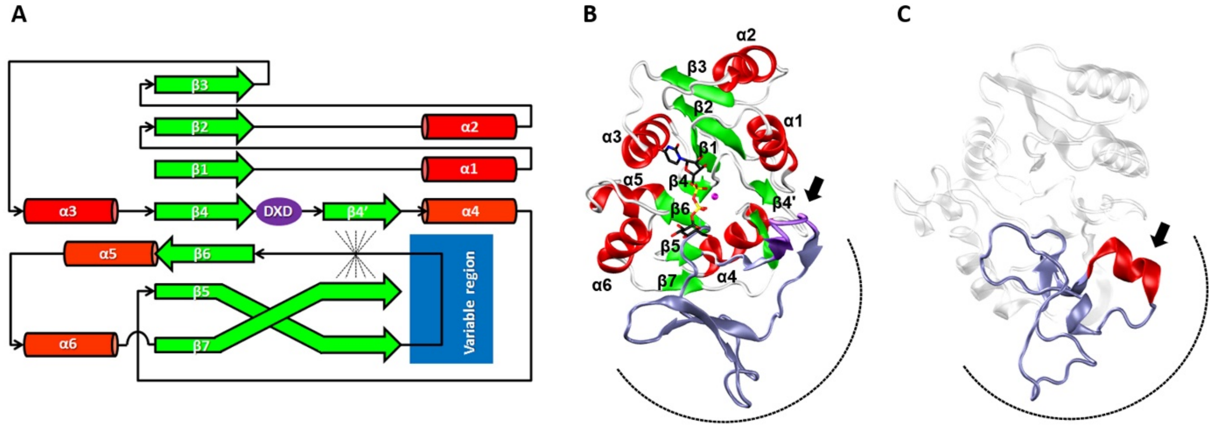

Figure S10. Hydropathic profile of the ful-length MG517 protein. Structural elements proposed for GTA consensus topology[2] are colored. Red: α-helixes. Green: β-sheets. Blue corresponds to the "Variable region". *VRαH*, α-helix under the variable region, proposed as a membrane adhesion element together h4 (amphipathic helix) due its hydropathic profile and topological position.

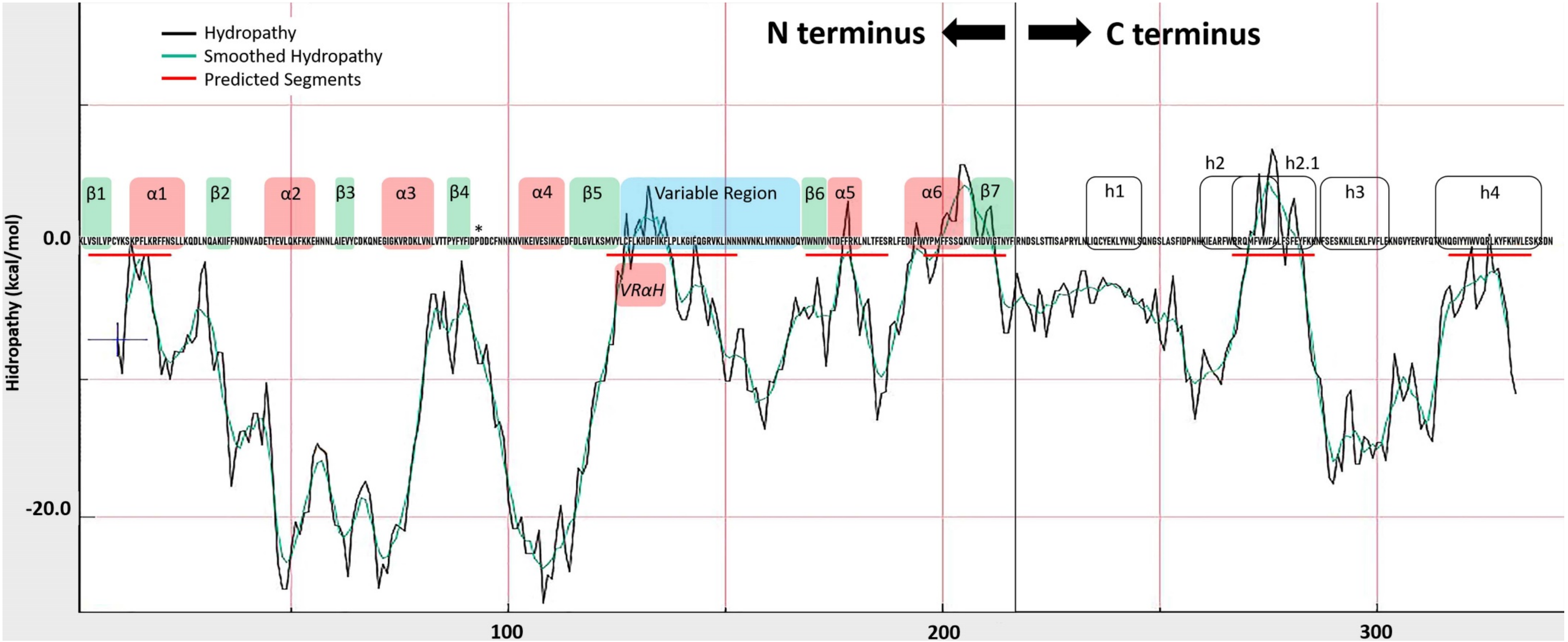

Figure S11. Hypothetical overall topology of full-length MG517 and the helices proposed for the membrane interaction. Green arrows: b-sheets, Red tubes: a-helixes, Purple circle: conserved DXD motif characteristic of glycosyl transferases, Blue square: structurally variable region and Red dashed-line: flexible loop characteristic of GT-A fold glycosyltransferases. The N-terminus domain (from β1 to α6) was reported by Romero-García *et al.*[2]. The C-terminus domain is proposed in this work based on secondary structure predictions, hydropathy profile analysis and molecular dynamics simulations. α-Helix 4 discussed in the text is labelled as "Amphipathic" in the cartoon.

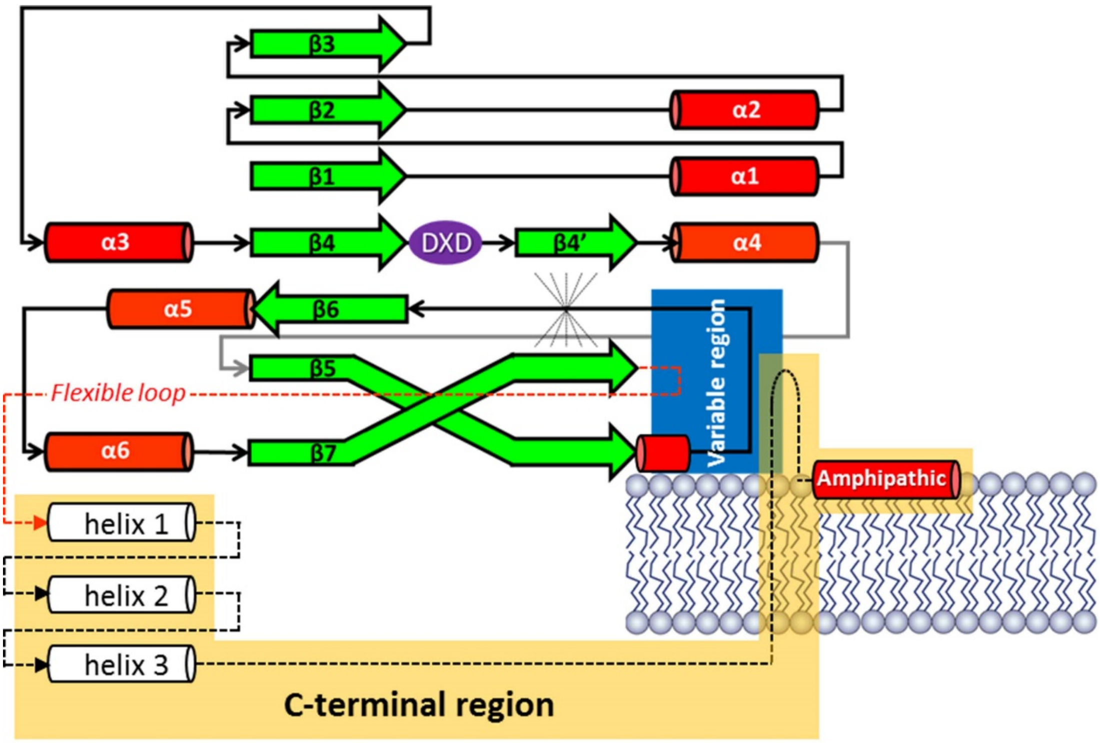

REFERENCES

1. Kim, D. E., Chivian, D. & Baker, D. Protein structure prediction and analysis using the Robetta server. *Nucleic Acids Res.* **32,** W526-31 (2004).

2. Romero-García, J., Francisco, C., Biarnés, X. & Planas, A. Structure-function features of a Mycoplasma glycolipid synthase derived from structural data integration, molecular simulations, and mutational analysis. *PLoS One* **8,** e81990 (2013).

3. Andrés, E., Martínez, N. & Planas, A. Glycolipid biosynthesis in mycoplasma genitalium: recombinant expression and characterization of a glycosyltransferase producing mono- and diglycosyldiacylglycerols in the plasma membrane. *J. Biol. Chem.* **286,** 35367–35379 (2011).

4. Vikström, S., Li, L., Karlsson, O. P. & Wieslander, A. Key role of the diglucosyldiacylglycerol synthase for the nonbilayer-bilayer lipid balance of Acholeplasma laidlawii membranes. *Biochemistry* **38,** 5511–20 (1999).
